# Matrix and cell phenotype differences in Dupuytren’s disease

**DOI:** 10.1186/s13069-016-0046-0

**Published:** 2016-06-29

**Authors:** Marike M. van Beuge, Evert-Jan P. M. ten Dam, Paul M. N. Werker, Ruud A. Bank

**Affiliations:** Department of Pathology & Medical Biology, University Medical Center Groningen, University of Groningen, Hanzeplein 1, 9713 GZ Groningen, The Netherlands; Department of Plastic Surgery, Department of Pathology & Medical Biology, University Medical Center Groningen, University of Groningen, Hanzeplein 1, 9713 GZ Groningen, The Netherlands

**Keywords:** Dupuytren’s disease, Fibroblast, Collagen biosynthesis, PCOLCE2

## Abstract

**Background:**

Dupuytren’s disease is a fibroproliferative disease of the hand and fingers, which usually manifests as two different phenotypes within the same patient. The disease first causes a nodule in the palm of the hand, while later, a cord develops, causing contracture of the fingers.

**Results:**

We set out to characterize the two phenotypes by comparing matched cord and nodule tissue from ten Dupuytren’s patients. We found that nodule tissue contained more proliferating cells, CD68-positive macrophages and α-smooth muscle actin (α-SMA)-positive myofibroblastic cells. qPCR analysis showed an increased expression of COL1A1, COL1A2, COL5A1, and COL6A1 in nodule tissue compared to cord tissue. Immunohistochemistry showed less deposition of collagen type I in nodules, although they contained more fibronectin, collagen type V, and procollagen 1. Lower collagen levels in nodule were confirmed by HPLC measurements of the Hyp/Pro ratio. PCOLCE2, an activator of BMP1, the main enzyme cleaving the C-terminal pro-peptide from procollagen, was also reduced in nodule. Cord tissue not only contained more collagen I, but also higher levels of hydroxylysylpyridinoline and lysylpyridinoline residues per triple helix, indicating more crosslinks.

**Conclusions:**

Our results clearly show that in Dupuytren’s disease, the nodule is the active disease unit, although it does not have the highest collagen protein levels. The difference in collagen type I deposition compared to mRNA levels and procollagen 1 levels may be connected to a decrease in procollagen processing.

**Electronic supplementary material:**

The online version of this article (doi:10.1186/s13069-016-0046-0) contains supplementary material, which is available to authorized users.

## Background

Any fibrotic process is an interplay between cells and matrix, with the matrix influencing cell proliferation, adhesion, and migration, and the cells influencing matrix composition and crosslinking [1]. This process is the ultimate consequence of a range of insults and can occur in virtually all organs of the body, with many differences and similarities between the organs. These differences may depend on the regenerative capacity of the organ or its capacity to compensate for the loss of function due to the build-up of extracellular matrix. All fibrotic diseases are characterized by the presence of myofibroblasts, a cell type containing abundant actin fibers. These cells are capable of producing and depositing large amounts of excess extracellular matrix, and their presence is therefore considered a main cause of fibrotic disease [2].

Dupuytren’s disease is a very common (prevalence 0.6 to 31.6 % [3]) fibroproliferative disease of the hand and fingers, which generally starts with the formation of a nodule in the palm of the hand, and progresses with the formation of a cord towards the fingers, which causes eventual contraction and the inability to extend the fingers. These two phenotypes commonly occur together in the same patient [4].

Previous studies have addressed the differences between cord and nodule and found that there are differences in contractility of isolated fibroblasts, with cells isolated from nodule being more contractile in vitro [5]. Furthermore, these cells express more α-smooth muscle actin [6]. A large majority of these studies however was performed on isolated, subcultured fibroblasts, which may grossly distort the phenotype of the cells [7]. Studies on complete tissue have shown that cord and nodule have a different ratio of expression of matrix metalloproteinases (MMPs) and tissue inhibitors of matrix metalloproteinases (TIMPs) [8, 9]. Older studies have reported differences in the type of extracellular matrix that is deposited in nodules or cords, with the former containing more fibronectin, laminin, collagen IV, and tenascin C, particularly in proliferative areas [10–12].

In a different classification, devised first by Luck [13], Dupuytren’s tissue has also been reported to contain several zones, which can be distinguished histologically as the proliferative, involutional, and residual zone. The proliferative zone is characterized by the presence of a large number of (myo)fibroblasts, the involutional zone contains large amounts of collagens and myofibroblasts, and the residual zone is relatively poor in cells.

Dupuytren’s disease displays the relatively rare phenomenon of two phenotypes (e.g., cord and nodule) at the same time in the same patient, with much discussion still going on in the field as to the origins of these tissues [14]. We investigated the differences between these phenotypes at a molecular level. Specifically, we wanted to address the question of whether the difference is a difference of the cell types present and/or a difference in the composition and amount of extracellular matrix. Differences in extracellular matrix (ECM) constituents and density between cord and nodule may account for pathogenesis of the two phenotypes and may influence current and future treatment options.

## Results

### Proliferation and cell types in cord versus nodule

**Fig. 1:**
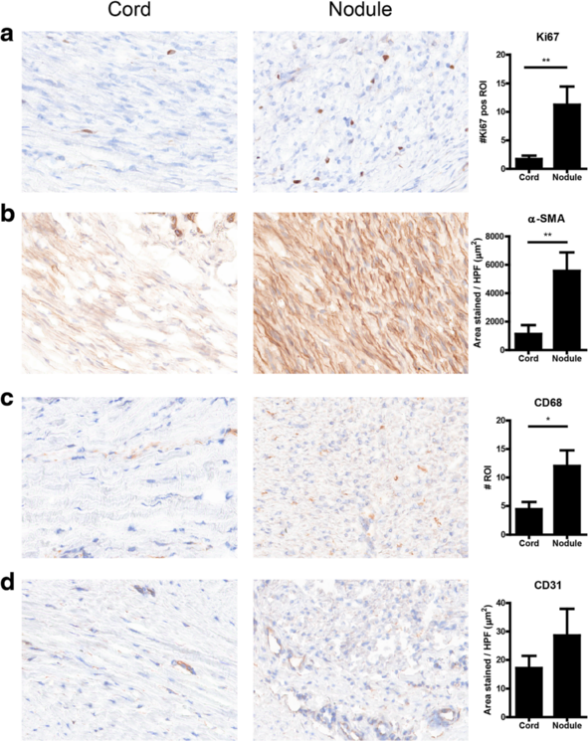
Cell types in cord and nodule tissue. **a** Representative pictures and quantification of Ki-67 expression in cord and nodule tissue from ten Dupuytren’s patients. **b** Representative pictures and quantification of α-smooth muscle actin expression in cord and nodule tissue from ten Dupuytren’s patients. **c** Representative pictures and quantification of CD68 expression in cord and nodule tissue from ten Dupuytren’s patients. **d** Representative pictures and quantification of CD31 expression in cord and nodule tissue from ten Dupuytren’s patients. *p < 0.05, **p < 0.01 as determined by Wilcoxon paired rank test

In order to determine whether the differences between cord and nodule are determined by the number, type, and activity of the cells present, we performed several immunohistochemical stainings. Staining for Ki-67, which is expressed in the nuclei of proliferating cells, showed that the number of proliferating cells was significantly higher in nodule tissue compared to cord tissue of the same patients (Fig. 1a). Furthermore, nodular tissue contained more α-smooth muscle actin (α-SMA), a marker for myofibroblasts (Fig. 1b), and more CD68-positive cells (Fig. 1c), which denotes mainly macrophages. We found no significant difference in the area of CD31-positive cells, indicating that there is no difference in the number of blood vessels between cord and nodule (Fig. 1d).

### Extracellular matrix production

To study the differences in the production of extracellular matrix between cord and nodule of Dupuytren’s patients, we first performed a gene expression analysis on extracellular matrix components and biosynthesis molecules. In total, we examined the expression of 44 genes; full results of this analysis can be found in Table 1; notable findings are discussed below.

**Table 1:** Median expression levels and fold change of all genes analyzed in Dupuytren’s nodule and matching cord tissue. *p* value determined by Wilcoxon paired rank test

Gene symbol	*N*	Mean cord	Mean nodule	Fold change nodule/cord	*p* value
ADAMTS14	8	0.0041	0.0124	2.988	0.0234
ADAMTS2	8	0.2375	0.2983	1.256	n.s.
ADAMTS3	6	0.0025	0.0032	1.321	n.s.
BGN	8	1.2588	1.5649	1.243	n.s.
BMP1	8	0.0424	0.0556	1.312	n.s.
COL1A1	8	4.2939	10.7051	2.493	0.0078
COL1A2	8	2.6263	4.7832	1.821	0.0078
COL3A1	8	3.7696	7.8217	2.075	n.s.
COL4A1	6	0.1868	0.1972	1.152	n.s.
COL5A1	8	0.3198	0.6450	2.017	0.0156
COL6A1	8	2.5967	4.1680	1.605	0.0234
COLGALT1	7	0.0386	0.0415	1.098	n.s.
CTSK	7	0.1254	0.1927	1.742	0.0313
DCN	8	0.7646	0.6807	0.890	n.s.
DDR1	6	0.0082	0.0068	0.768	n.s.
DDR2	8	0.0540	0.0421	0.780	0.0391
ELN	8	0.0959	0.1054	1.099	n.s.
FKBP10	8	0.1019	0.1440	1.414	0.0234
FMOD	8	0.1868	0.1334	0.714	n.s.
FN1	8	1.9316	3.4849	1.804	0.0078
LEPRE1	8	0.0140	0.0182	1.304	n.s.
LEPREL1	8	0.0023	0.0023	1.019	n.s.
LEPREL2	8	0.0154	0.0271	1.759	0.0156
LOX	7	0.0425	0.0603	1.597	0.0156
LOXL1	8	0.0400	0.0555	1.388	n.s.
LOXL2	8	0.1436	0.2461	1.714	n.s.
LOXL3	7	0.0067	0.0057	0.782	n.s.
LOXL4	8	0.0015	0.0009	0.574	n.s.
MMP1	4	0.0004	0.0003	0.839	n.s.
MMP13	6	0.0015	0.0023	1.302	n.s.
MMP14	8	0.3076	0.6230	2.026	n.s.
MRC2	7	0.1996	0.2784	1.378	0.0313
P4HA1	6	0.0094	0.0114	1.217	n.s.
P4HA2	5	0.0009	0.0012	1.363	n.s.
P4HA3	8	0.0171	0.0338	1.980	n.s.
P4HB	8	0.2390	0.3264	1.366	0.0234
PCOLCE	7	0.4790	0.5545	1.231	n.s.
PCOLCE2	7	0.0326	0.0107	0.224	0.0313
PLOD1	8	0.0730	0.0985	1.350	n.s.
PLOD2	8	0.0248	0.0380	1.533	n.s.
PLOD3	7	0.0423	0.0419	0.964	n.s.
SERPINH1	8	0.1895	0.2439	1.287	n.s.
SLC39A13	6	0.0538	0.0505	1.022	n.s.
TIMP1	8	0.1956	0.1915	0.979	n.s.

**Fig. 2:**
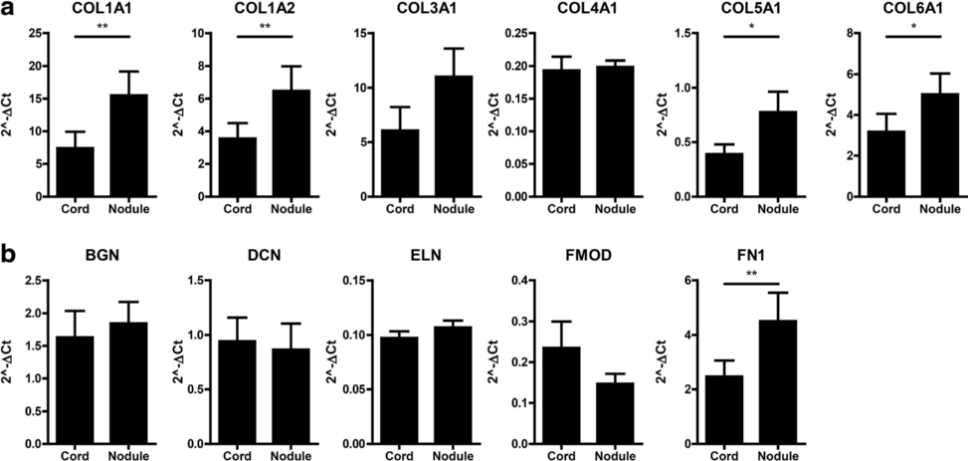
Extracellular matrix mRNA profile. **a** Relative mRNA expression of COL1A2, COL1A2, COL3A1, COL4A1, COL5A1, and COL6A1 in cord and nodule tissue from eight Dupuytren’s patients. **b** Relative mRNA expression of BGN, DCN, ELN, FMOD, and FN1 in cord and nodule tissue from eight Dupuytren’s patients. *p < 0.05, **p < 0.01 as determined by Wilcoxon paired rank test

We found a significantly higher expression of COL1A1, COL1A2, COL5A1, and COL6A1 messenger RNA (mRNA) in nodule tissue (Fig. 2a). There were no significant differences in gene expression of COL3A1 and COL4A1. In addition, five non-collagenous extracellular matrix molecules were studied; of these, there was only a significant difference in the expression of FN1, which was higher in nodule (Fig. 2b). There were no differences in the expression of BGN, DCN, ELN, and FMOD.

### Extracellular matrix deposition

**Fig. 3:**
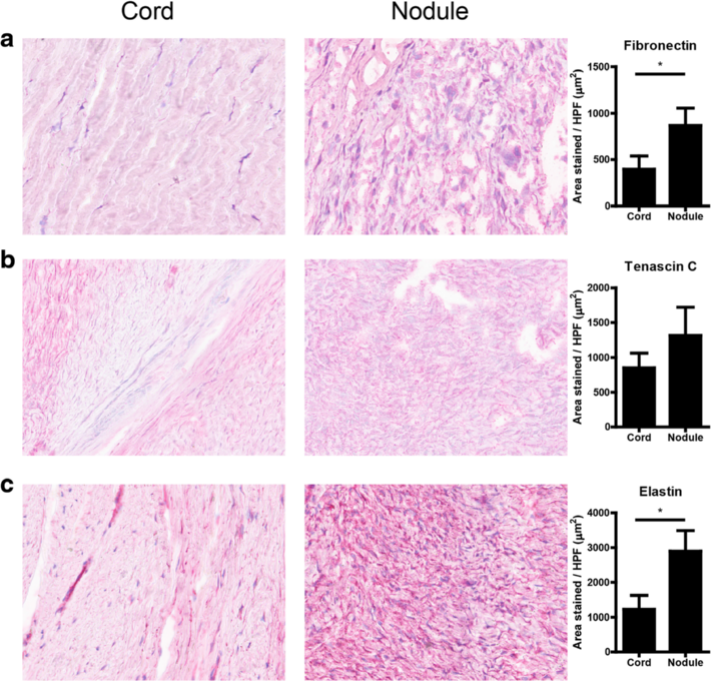
Non-collagenous extracellular matrix proteins. **a** Representative pictures and quantification of fibronectin expression in cord and nodule tissue from ten Dupuytren’s patients. **b** Representative pictures and quantification of tenascin C expression in cord and nodule tissue from ten Dupuytren’s patients. **c** Representative pictures and quantification of elastin expression in cord and nodule tissue from ten Dupuytren’s patients. *p < 0.05, **p < 0.01 as determined by Wilcoxon paired rank test

To study whether the differences we found in mRNA levels of ECM molecules also resulted in differences in the composition of the deposited ECM, we performed immunohistochemical analyses. We found significantly more fibronectin deposition in nodules compared to cords (Fig. 3a), whereas there was no difference in levels of tenascin C, which was earlier reported to be increased in nodules as well (Fig. 3b, [10]). However, we found more elastin present in nodules compared to cords (Fig. 3c).

**Fig. 4:**
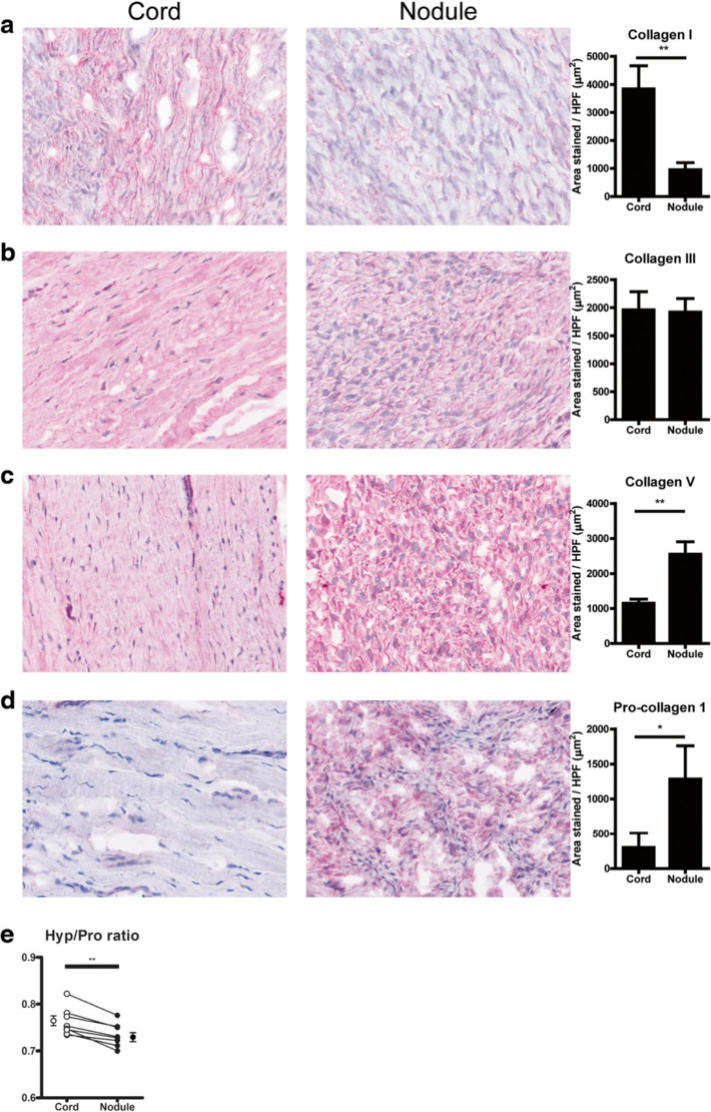
Collagenous extracellular matrix proteins. **a** Representative pictures and quantification of collagen I expression in cord and nodule tissue from ten Dupuytren’s patients. **b** Representative pictures and quantification of collagen III expression in cord and nodule tissue from ten Dupuytren’s patients. **c** Representative pictures and quantification of collagen V expression in cord and nodule tissue from ten Dupuytren’s patients. **d** Representative pictures and quantification of procollagen 1 expression in cord and nodule tissue from ten Dupuytren’s patients. **e** Graph showing the ratio between hydroxyproline and proline (Hyp/Pro) for matching cords and nodules from eight Dupuytren’s patients. *Dots* and *whiskers* on both sides represent means and SEM, respectively. *p < 0.05, **p < 0.01 as determined by Wilcoxon paired rank test

Analysis of collagen deposition showed that, in contrast to the mRNA data, collagen type I deposition in cord was significantly higher than in nodule (Fig. 4a), whereas there was no difference in the deposition of collagen type III (Fig. 4b), and a significant increase in collagen type V in nodule compared to cord (Fig. 4c). Contrary to collagen, a higher expression of procollagen type I is seen in nodule than in cord (Fig. 4d), which does correspond to the mRNA results. To verify our immunohistochemical data on collagen, we quantified the total amount of collagen in the tissues by measuring the Hyp/Pro ratio using HPLC. Increased collagen levels result in increased Hyp levels compared to Pro, giving rise to higher Hyp/Pro ratios [15]. Since collagen type I is the major collagen type in cords and nodules, one would expect (based on the collagen type I immunohistochemistry staining) in cord samples a higher Hyp/Pro ratio compared to nodule samples. This was indeed the case (Fig. 4e).

### Collagen I processing

**Fig. 5:**
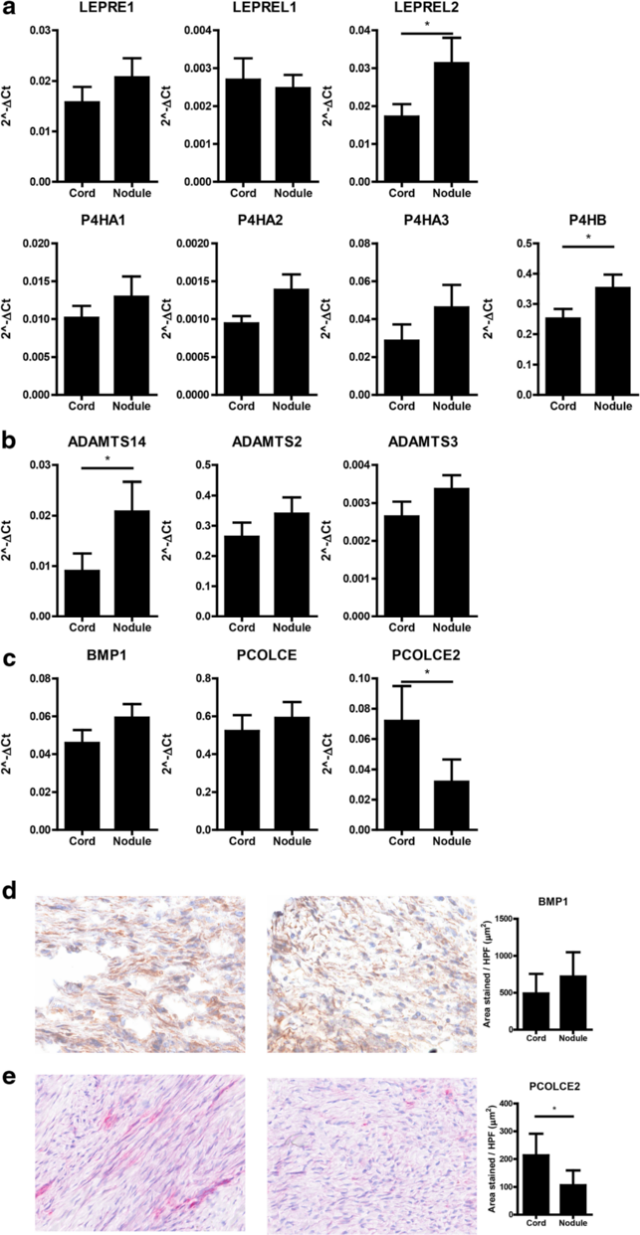
Collagen biosynthesis. **a** Relative mRNA expression of LEPRE1, LEPREL1, LEPREL2, P4HA1, P4HA2, P4HA3, and P4HB in cord and nodule tissue from eight Dupuytren’s patients. **b** Relative mRNA expression of ADAMTS2, ADAMTS3, and ADAMTS14 in cord and nodule tissue from eight Dupuytren’s patients. **c** Relative mRNA expression of BMP1, PCOLCE, and PCOLCE2 in cord and nodule tissue from eight Dupuytren’s patients. **d** Representative pictures and quantification of BMP1 expression in cord and nodule tissue from ten Dupuytren’s patients. **e** Representative pictures and quantification of PCOLCE2 expression in cord and nodule tissue from ten Dupuytren’s patients. *p < 0.05, **p < 0.01 as determined by Wilcoxon paired rank test

In view of the discrepancy between the mRNA levels of COL1A1 and COL1A2, which were higher in nodule and the observed deposition of collagen I which was higher in cord, we decided to further investigate this phenomenon. Analysis of mRNA expression of enzymes involved in collagen synthesis revealed an upregulation of LEPREL2, which has prolyl-3-hydroxylation activity, and P4HB, which catalyzes the formation of 4-hydroxyproline, both in nodule (Fig. 5a). Therefore, collagen synthesis may be slightly higher in nodule, depending on the relative activities of the different 3- and 4-hydroxylation enzymes. We then investigated procollagen processing and found that the expression of ADAMTS14 was significantly higher in nodule but very variable among patients. Levels of ADAMTS2 and ADAMTS3 were unchanged (Fig. 5b). There was no difference in the expression of BMP1, either at mRNA or protein level (Fig. 5c, d). However, we found a consistently lower mRNA expression of PCOLCE2, which activates BMP1, in nodules compared to cord (Fig. 5c), which was also confirmed at protein level by immunohistochemistry (Fig. 5e).

### Crosslinked collagen

**Fig. 6:**
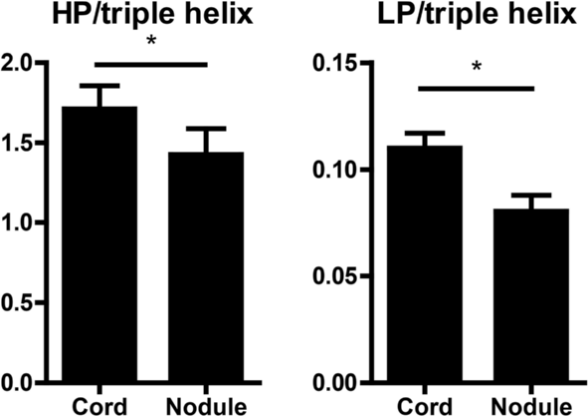
Collagen crosslinks. Number of hydroxylysylpyridinoline (HP) and lysylpyridinoline (LP) residues per triple helix. *p < 0.05, as determined by Wilcoxon paired rank test

The composition of the deposited collagen was examined by HPLC analysis of crosslinks, corrected for the amount of collagen present, expressed as number of hydroxylysylpyridinoline (HP) and lysylpyridinoline (LP) residues per triple helix. We found a significant increase of both types of crosslinks in cord tissue compared to nodule tissue (Fig. 6). In contradiction, of the main enzymes responsible for collagen crosslinking, we only found a significant difference in the expression of LOX, which was higher in nodule, whereas the expression of LOXL1-4 and PLOD1-3 was unchanged (Table 1).

## Discussion

In this study, we showed that there are considerable differences between cord and nodule at mRNA and protein level, both in cell types present as well as in the extracellular matrix composition. These differences are probably interdependent and together are responsible for the disease phenotype as it develops in patients. To our knowledge, this is the first study comparing cell types, matrix deposition, and the collagen biosynthesis pathway in cord and nodule tissue concurrently.

In a previous study [13], a proliferative stage was proposed to exist in Dupuytren’s disease, mainly in nodules, which coincides with our findings of higher Ki-67 expression in nodule. In an extension on this system, Lam et al. also found a correlation between collagen III expression and the stages proposed by Luck, with a higher proportion of collagen III in the proliferative stage [16]. In contrast, we did not find an increase in collagen III expression in nodule, which may be due to differences in the method of determination, i.e., immunohistochemistry in our study and a histochemical staining (Herovici’s staining) used by Lam et al. Additionally, differences may be caused by the heterogeneity of cord tissue, which we noticed especially in our immunohistochemical studies, where sometimes we found small nodule-like structures within the cords, as has also been reported by others [17]. These were however not large enough to obscure the differences between the two types of tissue, although they are undoubtedly partly responsible for the variation seen between the samples.

In accordance with previous papers examining tissue from Dupuytren’s patients, we found a significant amount of CD68-positive macrophages in nodule tissue [18]. The previous study also found significant levels of various growth factors and cytokines in Dupuytren’s tissue, although no comparison between cord and nodule was made. In view of the differences in composition of ECM between cord and nodule that we found, and the known capacity for ECM components, such as fibronectin, to serve as a repository for growth factors [19], we expect that the profile of associated growth factors will be very different in these types of tissue. Since nodule in addition is a more “active” type of tissue, where more remodeling might be occurring, a higher release of these growth factors is also to be expected.

We found that nodule tissue shows an active pro-fibrotic phenotype, with a high percentage of α-SMA-positive myofibroblasts, and some macrophages and high expression of fibronectin, procollagen 1, and collagen V. This profile bears a striking resemblance to the profile of active fibrogenic tissue as published by Blaauboer et al. [20]. In this study, the authors showed that the formation of new collagen in a mouse model for idiopathic lung fibrosis was correlated with an increased expression of type V collagen, elastin, tenascin C, lysyl oxidase, and Wnt-1 inducible signaling pathway protein 1 (WISP1). In our study, we found that in nodule, there is a high expression of type V collagen, elastin, tenascin C, and lysyl oxidase on mRNA and/or protein level. Furthermore, in a previous study, we have also found high expression of WISP1 in Dupuytren’s nodules [21]. An important difference is that in our study, the expression of the fibrotic phenotype was not accompanied by the deposition of collagen I, although we found a strong increase in COL1A1 and COL1A2 mRNA and procollagen 1 protein expression.

We propose that the apparent discrepancy between collagen mRNA and deposition is caused by a difference in the expression of collagen processing molecules between cord and nodule. After the synthesis of procollagen in the fibroblast, pro-peptides are cleaved off at the N- and C-terminal by ADAMTSs and BMP1, respectively (reviewed in [22, 23]). The cleavage of the C-terminal pro-peptide is essential for collagen fibril formation. Although we did not find any differences in BMP1 expression between cord and nodule at either mRNA or protein level, we did find a significantly lower expression of PCOLCE2 in nodule. BMP1 has many different functions in the cell, but only its effect on collagen processing is activated by PCOLCE(2) [24, 25]. At this moment, there is no consensus in literature about the relative contributions of the two different isoforms of PCOLCE, although they appear to have similar efficiency [26]. Intriguingly, in a study characterizing the phenotype of amniotic fluid-derived cells from fetuses with spina bifida, Hosper et al. found that these cells did not deposit any collagen, despite normal or increased levels of BMP1. These cells were then shown to have decreased levels of PCOLCE, PCOLCE2, and ADAMTSs [27]. In another study, PCOLCE2-null mice were shown to have decreased levels of collagen deposition after transverse aortic constriction [28]. Both studies give a clear indication of the essential role of these proteins in the eventual deposition of collagen by the cells.

In most studies into fibrotic conditions, the appearance of α-SMA-positive myofibroblasts and the excessive deposition of fibrillar collagen are seen as the hallmarks of fibrosis [2]. In this study, we did not find a correlation between the expression of α-SMA, which was higher in nodule, and the deposition of collagen, which was higher in cord. This may reflect the fact that all tissues were from patients in a later stage of Dupuytren’s disease, where α-SMA-positive myofibroblasts were originally present, but may have disappeared from the cord. Collagen, once deposited, may remain evident for a longer period than myofibroblasts, based on α-SMA expression. Whether these myofibroblasts have reverted to normal fibroblasts or been removed by apoptosis is not known, although lower cell numbers in cord make it likely that apoptosis must play a role. For this to be true, however, it presupposes that the cells previously present in cord were capable of depositing large amounts of collagen. These therefore cannot have been equivalent to nodule myofibroblasts, which do not deposit the collagen they produce.

Apart from the differences in composition of ECM, we also found qualitative differences in the collagen itself, since significantly, more crosslinks per triple helix were present in cord tissue than in nodule. Additionally, these HP and LP crosslinks are more difficult to degrade by endogenous MMPs, as described by van der Slot-Verhoeven et al. [29]. The higher crosslink levels in cord were in contradiction to a higher level of LOX mRNA in nodule, and no differences were found in the expression of LOXL1-4 and PLOD1-3. This pattern suggests that crosslinks in the cord collagen were formed previously, and in combination with the low levels of myofibroblasts and macrophages, is a profile reminiscent of previously fibrotic, now quiescent tissue, as was suggested in a previous study [17].

This may furthermore suggest that the extracellular matrix in the cord, once formed, is crosslinked and thereafter not remodeled further, with the cells responsible for the deposition of the matrix partly disappearing. The question remaining in this model is which cells are responsible for the contraction of the cord, leading to the patients’ inability to extend the fingers. A previous study has noted that nodule myofibroblasts possess an inherently greater contractile ability in vitro and suggested that these cells play a large role in contraction in patients as well [5].

In other forms of fibrosis, such as liver fibrosis, it has been shown that although few fibroblasts remain after partial resolution of fibrosis, the remaining cells are more prone to reacquire a fibrogenic phenotype after a new insult [30]. This mechanism might also be present in Dupuytren’s disease, where several comparative studies found that cord cells display an intermediate phenotype between nodule and normal fibroblasts [5, 6]. One of these studies also showed that although cord cells in normal culture show less α-SMA expression than nodule myofibroblasts, upon stimulation with TGF-β1, α-SMA expression was upregulated to the same extent as in nodule myofibroblasts [6], suggesting that they retain fibrogenic capacity.

If nodule is indeed the more active fibrogenic tissue, and if cord remains prone to reactivation, these factors have to be taken into account in one of the current problems with the treatment of Dupuytren’s disease, which is the high rate of recurrence. Recent papers suggest a slightly lower durability of the result after collagenase treatment, in which only the cord is targeted, compared to fasciectomy, in which both nodule and cord are removed [31]. Percutaneous needle fasciotomy, in which only the cord is disrupted, was also reported to have higher rates of recurrence than fasciectomy [32]. Without examination of tissue from patients with recurrent Dupuytren’s disease, however, it is currently not possible to say whether reactivation of cord tissue occurs and whether this is initiated by cord fibroblasts reactivated by the procedure, by myofibroblasts from the nodule or by a different process.

## Conclusions

We have found that nodule is the more pro-fibrotic tissue in Dupuytren’s disease, characterized by an RNA profile consistent with fibrogenesis, but with lower levels of actual collagen I deposition, possibly caused by an abnormal collagen biosynthesis, as evidenced by a lower expression of PCOLCE2. Cord tissue contains more collagen I, which is additionally more heavily crosslinked. These differences should be taken into account when deciding the optimal treatment for Dupuytren’s disease.

## Methods

### Ethics statement

Tissue samples were obtained following informed written consent and approval of the Medical Ethics Committee of the University Medical Centre Groningen (2007/067), in line with the Declaration of Helsinki.

### Primary tissues

Dupuytren’s nodules and cords were obtained from patients undergoing limited fasciectomy or dermofasciectomy in the University Medical Centre Groningen. Tissue from ten patients in total was used; nodules and cords of eight patients were analyzed by low density array and crosslink analysis; nodules and cords of ten patients were analyzed using immunohistochemistry.

### Gene expression analysis

The expression of genes known to be involved in collagen biosynthesis and homeostasis was determined with a custom-made microfluidic card-based low-density array (Additional file 1: Table S1; Applied Biosystems, Foster City, CA). This enables accurate measurement of gene expression levels of 44 simultaneously, using a Taqman probe system. RNA was isolated from tissue using the RNeasy Fibrous Tissue Mini Kit (Qiagen), according to the manufacturer’s instructions and quantified using a NanoDrop-1000 spectrophotometer (NanoDrop Technologies, Wilmington, DE). Reverse transcription was carried out using the first-strand complementary DNA (cDNA) synthesis kit (Fermentas, St. Leon-Rot, Germany). For each sample, 100 ng cDNA was diluted in 50 μL of distilled water and mixed with 50 μL of TaqMan PCR master mix (Applied Biosystems). Standard recommended PCR protocols were performed (50 °C for 2 min, 95 °C for 10 min, and the next two steps were repeated for 40 cycles: 95 °C for 12 s and 60 °C for 1 min) using the ViiA™ 7 Real-Time PCR System (Applied Biosystems). Threshold cycle numbers higher than 35 were set to 35 and considered not detectable. Patients were removed from the analysis if there was no detectable expression in both cord and nodule; numbers of patients included per gene are shown in Table 1. Gene expression was calculated normalized to the geometric mean of four reference genes (β-actin, β2-macroglobulin, GAPDH, and YWHAZ).

### Immunohistochemistry

Tissue for staining was stored at −80 °C and cut into 5 μm cryosections. The sections were air-dried for 30 min and fixated for 10 min in acetone. Washing and blocking of aspecific binding sites, endogenous peroxidases, and endogenous biotin was performed according to standard procedures. The sections were incubated for 60 min at RT with primary antibody (see Additional file 2: Table S2), and with species specific HRP- or biotinylated secondary antibodies (DAKO and Southern Biotech). Stainings were visualized using VECTOR Red Alkaline Phosphatase (AP) Substrate or ImmPACT DAB Peroxidase (SK5100 or SK4105, respectively, Vector Laboratories, Burlingame, CA) according to the manufacturer’s instructions. All immunohistochemical stainings were counterstained with hematoxylin (Merck, Darmstadt, Germany) and mounted in Kaiser’s glycerin-gelatin (Merck).

### Quantification of stainings

Immunohistochemical stainings were evaluated using a Leica DM 2000 microscope. For morphometric quantification of immunohistochemistry, five representative photomicrographs at 40× magnification were taken per tissue section, using a Multispectral Imaging Camera (Perkin Elmer, Cambridge, UK). Photomicrographs were analyzed using Nuance 3.0 software (Perkin Elmer). Stained areas were quantified and expressed as square micrometer per high power field (μm^2^/HPF).

### Crosslink analysis

Hydroxyproline (Hyp), proline (Pro), and the crosslinks hydroxylysylpyridinoline (HP) and lysylpyridinoline (LP) were measured in acid hydrolysates of unreduced samples by reversed-phase high-performance liquid chromatography [33, 34]. The Hyp/Pro ratio was used as a measure to estimate the ratio of collagen towards non-collagenous proteins: the lower the Hyp/Pro ratio, the more non-collagenous proteins are present in the tissue [15]. The crosslinks HP and LP are expressed as the total amount of residues per collagen molecule, assuming 300 Hyp residues per triple helix.

### Statistics

Statistical analysis was performed using GraphPad Prism 5.0 using a Wilcoxon paired rank test. In all analyses, *p* < 0.05 was considered to be statistically significant.

## Abbreviations

α-SMA, α-smooth muscle actin; AP, alkaline phosphatase; ECM, extracellular matrix; HP, hydroxylysylpyridinoline; HPF, high power field; Hyp, hydroxyproline; LP, lysylpyridinoline; MMP, matrix metalloproteinases; Pro, proline; TIMP, tissue inhibitor of matrix metalloproteinase; WISP1, Wnt-1 inducible signaling pathway protein

## Additional files

Additional file 1: Set up of custom made microfluidic card-based low density array (Applied Biosystems, Foster City, CA). (DOCX 14 kb) Additional file 2: Primary antibodies used. (DOCX 12 kb)
