# Supplementary material for: Matrix and cell phenotype differences in Dupuytren’s disease
Source: Fibrogenesis Tissue Repair. 2016 Jun 29;9:9. doi: 10.1186/s13069-016-0046-0 (PMC4928329; doi:10.1186/s13069-016-0046-0)
Supplement: Additional file 1: — Set up of custom made microfluidic card-based low density array (Applied Biosystems, Foster City, CA). (DOCX 14 kb) [file 13069_2016_46_MOESM1_ESM.docx]

Suppl. Table 1: Set up of custom made microfluidic card-based low density array (Applied Biosystems, Foster City, CA).

| Gene symbol | Gene name | Assay ID |
| --- | --- | --- |
| PLOD1 | procollagen-lysine, 2-oxoglutarate 5-dioxygenase 1 | Hs00609368_m1 |
| PLOD2 | procollagen-lysine, 2-oxoglutarate 5-dioxygenase 2 | Hs00168688_m1 |
| PLOD3 | procollagen-lysine, 2-oxoglutarate 5-dioxygenase 3 | Hs00153670_m1 |
| P4HA1 | prolyl 4-hydroxylase, alpha polypeptide I | Hs00914594_m1 |
| P4HA2 | prolyl 4-hydroxylase, alpha polypeptide II | Hs00188349_m1 |
| P4HA3 | prolyl 4-hydroxylase, alpha polypeptide III | Hs00420085_m1 |
| P4HB | prolyl 4-hydroxylase, beta polypeptide | Hs00168586_m1 |
| LEPRE1 | leucine proline-enriched proteoglycan (leprecan) 1 | Hs00223565_m1 |
| LEPREL1 | leprecan-like 1 | Hs00216998_m1 |
| LEPREL2 | leprecan-like 2 | Hs00204607_m1 |
| GAPDH | glyceraldehyde-3-phosphate dehydrogenase | Hs99999905_m1 |
| B2M | beta-2-microglobulin | Hs00187842_m1 |
| LOX | lysyl oxidase | Hs00942480_m1 |
| LOXL1 | lysyl oxidase-like 1 | Hs00935937_m1 |
| LOXL2 | lysyl oxidase-like 2 | Hs00158757_m1 |
| LOXL3 | lysyl oxidase-like 3 | Hs01046945_m1 |
| LOXL4 | lysyl oxidase-like 4 | Hs00260059_m1 |
| SERPINH1 | serpin peptidase inhibitor, clade H (heat shock protein 47), member 1, (collagen binding protein 1) | Hs00241844_m1 |
| ADAMTS2 | ADAM metallopeptidase with thrombospondin type 1 motif, 2 | Hs00247973_m1 |
| ADAMTS3 | ADAM metallopeptidase with thrombospondin type 1 motif, 3 | Hs00610744_m1 |
| ADAMTS14 | ADAM metallopeptidase with thrombospondin type 1 motif, 14 | Hs00365506_m1 |
| BMP1 | bone morphogenetic protein 1 | Hs00241807_m1 |
| PCOLCE | procollagen C-endopeptidase enhancer | Hs00170179_m1 |
| PCOLCE2 | procollagen C-endopeptidase enhancer 2 | Hs00203477_m1 |
| COL1A1 | collagen, type I, alpha 1 | Hs00164004_m1 |
| COL1A2 | collagen, type I, alpha 2 | Hs00164099_m1 |
| COL3A1 | collagen, type III, alpha 1 | Hs00943809_m1 |
| COL4A1 | collagen, type IV, alpha 1 | Hs00266237_m1 |
| COL5A1 | collagen, type V, alpha 1 | Hs00609088_m1 |
| COL6A1 | collagen, type VI, alpha 1 | Hs01095585_m1 |
| FN1 | fibronectin 1 | Hs00365052_m1 |
| ELN | elastin | Hs00355783_m1 |
| FKBP10 | FK506 binding protein 10, 65 kDa | Hs00222557_m1 |
| SLC39A13 | solute carrier family 39 (zinc transporter), member 13 | Hs00378317_m1 |
| YWHAZ | tyrosine 3-monooxygenase/tryptophan 5-monooxygenase activation protein, zeta polypeptide | Hs03044281_g1 |
| ACTB | actin, beta | Hs01060665_g1 |
| DCN | decorin | Hs00370385_m1 |
| BGN | biglycan | Hs00959143_m1 |
| FMOD | fibromodulin | Hs00157619_m1 |
| MMP1 | matrix metallopeptidase 1 (interstitial collagenase) | Hs00899658_m1 |
| MMP13 | matrix metallopeptidase 13 (collagenase 3) | Hs00233992_m1 |
| MMP14 | matrix metallopeptidase 14 (membrane-inserted) | Hs00237119_m1 |
| TIMP1 | TIMP metallopeptidase inhibitor 1 | Hs99999139_m1 |
| CTSK | cathepsin K | Hs00166156_m1 |
| DDR1 | discoidin domain receptor tyrosine kinase 1 | Hs00233612_m1 |
| DDR2 | discoidin domain receptor tyrosine kinase 2 | Hs00178815_m1 |
| COLGALT1 | collagen beta(1-O)galactosyltransferase 1 | Hs00430696_m1 |
| MRC2 | mannose receptor, C type 2 | Hs00195862_m1 |
