# Supplementary material for: Matrix and cell phenotype differences in Dupuytren’s disease
Source: Fibrogenesis Tissue Repair. 2016 Jun 29;9:9. doi: 10.1186/s13069-016-0046-0 (PMC4928329; doi:10.1186/s13069-016-0046-0)
Supplement: Additional file 2: — Primary antibodies used. (DOCX 12 kb) [file 13069_2016_46_MOESM2_ESM.docx]

Suppl. Table 2: Primary antibodies used.

| Name | Manufacturer | Cat.no. | Dilution | Secondary Ab |
| --- | --- | --- | --- | --- |
| α-SMA | DAKO | M0851 | 1:100 | HRP |
| BMP1 | Sigma | HPA014572 | 1:100 | HRP |
| CD31 | Santa Cruz | sc-1506 | 1:100 | HRP |
| CD68 | DAKO | M0718 | 1:1000 | HRP |
| Collagen I | Abcam | ab6308 | 1:5000 | Biotin |
| Collagen III | Abcam | ab6310 | 1:5000 | Biotin |
| Collagen V | Abcam | ab7046 | 1:400 | Biotin |
| Elastin | Abcam | ab21610 | 1:100 | Biotin |
| Fibronectin | Abcam | ab2413 | 1:100 | Biotin |
| Ki-67 | Abcam | ab16667 | 1:200 | HRP |
| PCOLCE2 | Sigma | HPA013203 | 1:50 | Biotin |
| Procollagen 1 | Abcam | ab64409 | 1:250 | Biotin |
| Tenascin C | Abcam | ab6393 | 1:100 | Biotin |
